# Toward more equal and mutual human-pet relations: Insights and possible solutions based on social psychological theories

**DOI:** 10.3389/fvets.2022.1009267

**Published:** 2022-11-09

**Authors:** Catherine E. Amiot, Laurence Santerre-Bélec

**Affiliations:** ^1^Department of Psychology, Université du Québec à Montréal, Montréal, QC, Canada; ^2^Department of Didactics, Université du Québec à Montréal, Montréal, QC, Canada

**Keywords:** human-pet relations, social power, mutualism, social psychological theories, intergroup relations, self-determination

## Abstract

Human-pet relations are imbued with power imbalances, with many pets depending on humans for food and water, shelter, health care, and sheer survival. A majority of people report loving their pets and consider them to be integral family members; however, the care provided to pets varies widely and can be, in some cases, suboptimal. Yet, building more equal relations between humans and their pets could provide benefits to both parties. To achieve this increased equality and mutuality, the current paper proposes theory-based solutions. Specifically, and building on established social psychological theories, namely theories of intergroup relations and of human motivation, the current paper identifies both social and relational factors which, if socially and individually promoted, could trigger more equal and possibly mutually beneficial relationships with pets. We provide concrete examples illustrating how these factors can be maximized and promoted.

“A friendly relationship seems to be one in which something else exists not for us but for its own sake. That's the gold standard, whether it's a fellow human, a future child, or another living thing” [(1) p. 194].

“With great power comes great responsibility” (2).

Human relationships with pets are ubiquitous and normative in many occidental countries (3–5). These relationships have spanned different epochs and cultures (6) and can have deep and consequential outcomes for us humans. For example, pets have the capacity to elicit interactions with fellow humans (7); they can encourage physical activity among certain owners (8) and promote the “feel-good” hormones (9). Our human tendency to have pet dogs (or not) even has a genetic basis (10). While a majority of people love their pets and consider them as integral family members (5, 11), variability exists in terms of how pets are treated by humans, and the extent to which the pets' own needs are satisfied (5, 12). Furthermore, human-pet relations are imbued with clear power differentials: Pets generally depend on us for food and water, shelter, health care, and sheer survival (13). The current paper aims to provide theoretically-based solutions for how to lessen these power differentials, and to build more equal and mutual relations between humans and pets.

Acknowledging these power differences and the responsibility that comes with our human power over pets, as well as providing pets with more control and autonomy in their daily lives—within realistic constraints –, is important for the following reasons: First, doing so is likely to maximize the welfare of pets. Indeed, feeling autonomous and in control has been tied to improved wellbeing and reduced stress among both humans and other animals (14, 15). Second, pets whose welfare is maximized and whose needs are cared for are less likely to display problematic behaviors and to be relinquished (16, 17). Third, creating more equal relations between humans and pets could reverberate positively onto human-pet relations, and have benefits for humans as well. Indeed, emerging research is revealing the interconnections that exist between humans' and pets' wellbeing [e.g., in terms of long-term stress levels (18)]. Hence, and in line with a One Health perspective, the wellbeing of one (i.e., pet) species could have direct repercussions for the wellbeing of the other (i.e., human) (19).

It should be noted that the solutions presented herein do not aim to build completely equal relations between people and animals, in an absolute sense (e.g., in terms of equal societal rights and obligations, or in terms of access to the same resources); rather, the underlying goal is to provide *more* power to pets, in daily life and within realistic limits, relative to the power and control that some pets may currently have. By mutual relations, we mean relations where both humans and pets benefit from the relationship (6, 20–22). And while other research has focused on the factors that explain why humans prioritize their own species rather than others (23), the current paper specifically focuses on solutions for attenuating this tendency, specifically within human-pet relations. The solutions presented in this article pertain to, and aim to be applicable to, a variety of pet species; nevertheless, many examples pertain specifically to dogs.

Theoretically, the solutions put forward in this paper to achieve more equal and mutual relations between humans and their pets build on social psychological theories. Table 1 presents an overview of these social psychological theories and their main concepts and principles, as well as the solutions based on these. Table 1 also presents these theories and concepts in the order in which they are covered in the article. The first set of theories—i.e., theories of intergroup relations—accounts for the intergroup and power dynamics involved in human-pet relations, as well as the societal and macroscopic factors that could shape these relations and promote more equality and mutuality. The second theory—i.e., self-determination theory—accounts for the motivational processes and relational factors that are likely to yield more positive and mutual human-pet relations. Bringing together these theoretical models hence represents a useful integration to achieve both social- and individual-level changes.

**Table 1:** Overview of the theoretically-based solutions proposed for achieving more equal and mutual human-pet relations.

**Social psychological theory**	**Concept/principle**	**Solution**
Intergroup Contact	Promoting equal group status within the contact situation	To raise the status of pets relative to humans, strategically remind people of how pets are generally viewed as friends and as integral members of the family.
	Creating common goals	Structuring daily activities such that they involve reaching mutually beneficial and fun goals for the pet and the human (exercising, socialization).
	Promoting intergroup cooperation	Engaging in activities in which both the human and the animal are interdependent and form a team, and where each team member's unique capacities and strengths are brought together.
	Authorities, law, or customs support the contact	Authorities can structure, support, and promote contact with pets in public areas.
	Promoting positive affective processes (e.g., empathy, perspective taking)	Take into account the pet's own perspectives, histories, and specificities; Attend to the pet's specific needs.
	Promoting cognitive processes (i.e., knowledge about one's pet)	Learn about one's pet:, their species-specific particularities, and their individual preferences.
Social Identity Theory	Shifts in social power	Pets can be given more decision-making power; humans and social structures can accommodate to the needs of pets
	Social norms	Promoting and making salient dynamic (i.e., changing) social norms that increasingly recognize pets' needs and sentience
	Social identification	Developing greater solidarity with animals; putting forward the social identities that we share with pets (i.e., same family, same community)
Self-determination theory	Need for autonomy	Providing pets with opportunities for decision-making; clicker training to reinforce spontaneously emitted behaviors; gathering pet consent prior to interactions; identifying our pets' own preferences, strengths, and limits through observation and awareness; develop two-way communication by using different training tools (“talking buttons”, pet doorbell)
	Need for competence	Organizing foraging activities using food; using interactive food bowls; providing informational and positive feedback (i.e., using clicker training, by setting progressive, achievable and realistic goals); use of play to promote learning and the mastery of new abilities
	Need for relatedness	Ensuring that one's pet will be cared for unconditionally, demonstrating attention, love, and affection to our pet; showing our pet respect for whom he or she is; realizing how paying attention to our pet is beneficial for us
	Internalization	The use of intermittent reinforcement schedules and of secondary reinforcers to promote the internalization of our human-centric behaviors among pets

## Human-pet relations as imbued with power: Recognizing the elephant in the room

According to some historical accounts, human-pet relations have required that we humans use power over animals in order to control them and to achieve our goals (24), creating an unjust relationship between pets and humans (25). Wynne further proposed that people occupy a status of “super-dominance” over their pet dogs, given humans' high level of control over the resources that dogs need (26). This perspective contrasts with a view whereby pets are considered to be integral members of the family and friends to humans. Yet, perceiving the human-pet relation as one based solely on friendship may make it more difficult to recognize the potential for humans to exploit and neglect their pets, and misuse their power. Indeed, according to Benz-Schwazburg and colleagues: “While ethical debates have convincingly pointed to human responsibilities, for example in the case of farm animals and lab animals, companion animals are often not so clearly seen as animals which we “use,” objectify, or instrumentalize, maybe because the term “companion” indicates to some degree a mutual relationship rather than an exploitative one” [(13) p2]. We hence need to recognize the power imbalance that exists within human-pet relations and use this power responsibly.

In the ethological literature, different definitions of power, or more specifically, dominance, have been put forward. Dominance in this literature refers to the tendency for certain individuals in a social group to have at least partially consistent preferential access to valued and limited resources, such as shelter, food, and sexual partners (27, 28). When alluding to the notion of dominance, Frans de Waal (29) further proposed that alpha males and females, who occupy the highest ranks in primate hierarchies, need to demonstrate vigor, force, and unity, but also generosity and caring behaviors toward the other members of the group to achieve their higher-ranking positions. In this sense, dominance involves a high degree of responsibility and empathy, it can co-occur with cooperative and affiliative behavior, and it does not necessarily imply aggression. Focusing specifically on pets' behaviors, veterinary behaviorists concur in viewing dominance as a social status that may be attained without displaying aggressive behaviors (30).

These ethological conceptualizations of dominance partly align with definitions of social power used in the social psychological literature, whereby power can be defined as the degree of control members of one social group have upon their own fate and that of other groups [i.e., outgroups (31)], including the control over valued resources (32, 33). Such resources can be of a material (e.g., goods and money) or social nature [e.g., access to information and decision-making opportunities (34)]. In essence, having power enables more choice and degree of freedom (35). Importantly, and in line with the ethological literature, having high social power does not automatically imply that this power will be used in a self-interested or abusive manner; instead, power can be used in a socially responsible way (36). While power can take place in interpersonal relationships (32, 34), an intergroup—or interspecies—approach to social power will be adopted herein given the nature of human-pet relations. Indeed, the relations taking place between pets and humans involve members of different categories or groups (i.e., species) who interact together. The questions become: How can we, humans, use this social power responsibly, specifically in the context of our relationships with pets? And how can we build more equal relations between humans and pets, to our mutual benefit? To provide answers to these questions, we turn to two important and widely used sets of theories in social psychology, namely theories of intergroup relations and of human motivation.

## Theories of intergroup relations

Theories of intergroup relations are useful to understand human-animal relations in general (23, 37, 38), and human-pet relations in particular (39, 40). These theories also explicitly account for the power dynamics that exist between social groups (41), including between the members of different species (38, 42). Importantly, and given that they identify the social and individual factors which can yield more harmonious intergroup relations between different social groups, these theories also contain the seeds of possible solutions for promoting more equal and mutual human-pet relations in particular. We cover two specific theories of intergroup relations that appear particularly applicable and relevant to understanding power dynamics in human-pet relations: Intergroup contact theory and social identity theory. The main concepts and principles of these theories are presented in Table 1.

### Intergroup contact theory

Intergroup contact theory puts forward intuitive yet powerful propositions to explain when and why intergroup relations are more likely to be harmonious (43–45). According to this theory, promoting contact between the members of different social (e.g., cultural, national, religious) groups can improve the relations that will take place between these group members (44). Meta-analyses have confirmed this contention (46, 47). Furthermore, friendships formed with the members of an outgroup (i.e., intergroup friendships), as one specific type of intergroup contact, can also foster more positive intergroup attitudes toward this outgroup more generally (47). Because human-pet relations can be seen as a particular type of intergroup friendship, prior research has applied intergroup contact theory to the realm of human-pet relations specifically. This work has shown that the greater people's history of positive and frequent contact with pets, the lower their feelings of anxiety toward animals more generally; such lower anxiety, in turn, predicted more positive attitudes toward animals (39). Experimental research has also found that just imagining a positive contact with a pet dog [i.e., an imagined type of intergroup contact (48)] increased people's behavioral intentions to act on behalf of other animals (40).

Of course, contact with outgroup members is not always positive; in the intergroup contact literature, negative contact with an outgroup member has been found to deteriorate attitudes toward that outgroup as a whole (49–51). In the realm of human-pet relations, negative interactions with pets can be traumatic and impede these relations [e.g., dog bites (52)]. Contact with pets should hence be structured to avoid such negative contacts from taking place, or else all efforts at promoting more mutuality between humans and pets could backfire. In addition, creating situations of contact between the members of different social groups that respect certain conditions may yield even greater intergroup benefits (i.e., “when” will this contact be beneficial), in line with Allport's (43) optimal conditions for intergroup contact (45). These conditions appear particularly applicable to human-pet relations and useful to building more equal and mutual relations between people and their pets. Such *optimal conditions* for beneficial intergroup contact are as follows:

1. Equal group status within the contact situation: When members of different social groups interact together as “equals,” this intergroup contact should be more likely to lead to harmonious intergroup relations. In the context of human-pet relations, this condition for mutually beneficial contact could be met by attributing pets a social status that is more at par with humans,” rather than structuring this relationship such that it is highly hierarchical. Because pets are widely considered as “friends” and integral members of the family, it becomes logical, on this basis, to attribute them a more equal status. Reminding people of these commonly shared social beliefs could be used as a strategy to promote a more equal status for pets relative to humans.
2. Creating common goals: Intergroup contact that is structured such that members of different social groups rely on each other to achieve their shared goals should also yield more positive intergroup relations. This condition is more concrete and likely to be accessible to pet owners; it could be achieved by structuring daily activities such that they involve reaching mutually beneficial and fun goals, both for the pet and the human, for example: exercising/walking, going outside, playing games, organising foraging activities, visiting/welcoming a relative or friend at home dog. It should be noted that many of these activities can take place with a diversity of pet species (e.g., dogs, horses, cats, birds).
3. Promoting intergroup cooperation: Similarly, when members of different social groups engage in activities that require the cooperation of both groups, such contact is also more likely to ameliorate intergroup relations. A typical example in the context of human-pet relations involves participating in sports in which both the human and the animal are interdependent and form a team, and where each team member”s unique capacities and strengths are brought together (e.g., tracking or agility with dogs, endurance sports with horses).
4. Support of authorities, law, or custom: Contact which is supported by institutions and authorities is another condition that should yield more beneficial intergroup relations according to the intergroup contact literature. Contact with pets can be structured, supported, and promoted by authorities (e.g., governments, schools) through a diversity of measures. For example, allowing the presence of pets in specific and agreed-upon public areas (e.g., outdoor seating of restaurants, specific stores, public transport, trails within parks, pet-friendly workplaces), should contribute to the development of more positive attitudes toward pets, and to the establishment of more mutually beneficial relations. Interestingly, by allowing access to pets in these public places, pets may become better socialized and calmer; this, in turn, could contribute to fueling people's more positive perceptions of pets more generally, hence promoting a virtuous spiral.

In addition to these optimal conditions for contact, intergroup contact exerts its benefits through a number of *mediating* mechanisms (i.e., “why” is contact beneficial). Prior intergroup contact research has revealed a particularly clear role for the emotional mediators; during intergroup encounters, these mediators reduce negative affect (e.g., intergroup anxiety) and promote positive affects (i.e., empathy and perspective taking) (53, 54). When applying these principles and observations to the realm of human-pet relationships *per se*, positive interactions with pets could directly contribute to attenuating the negative emotions of fear, insecurity, and uncertainty about other species of animals (39), and instead build a feeling of trust and connection with them.

On these bases, human-pet contact could be structured so that it directly promotes the beneficial mechanisms of empathy and perspective taking. In this vein, different researchers have argued in favor of taking the pets' own perspectives, histories, and specificities to build more mutual human-pet relations (20, 55–57). Concretely, empathy and perspective taking could be maximized in the context of human-pet relations as one learns to care for and attend to their pet's own needs (rather than by adopting an anthropocentric view of one's pet's needs and preferences). One concrete example of this would involve choosing a diet for one's pet that aligns with the pet's specific dietary needs (rather than basing a pet's diet on the owner's own dietary preferences or habits).

While the intergroup contact literature has found that the more cognitive mediators (e.g., knowledge about another social group) play a less important role compared to the affective ones (e.g., empathy) in promoting more harmonious intergroup relations, such cognitive processes could still play a beneficial role in fostering more positive and mutual human-pet relations *per se*. For example, learning about one's pet and gathering knowledge about the particularities of their species, and also about one's pet own individuality and personality, could be particularly beneficial to building a more mutual human-pet relation (58, 59). Indeed, when building enrichment activities that promote the expression of an animal's own specific skills, it is necessary to know the behavioral repertoire of their species, as well as the preferences of the individual animal (60, 61). This knowledge is likely to promote a sense of familiarity with our pet, as well as increase our capacity to understand how our pet feels, thinks, and communicates. Such knowledge, although cognitive, could contribute to facilitating human-pet interspecies communication, and possibly yield more positive and mutually beneficial human-pet relations (62, 63). Indeed, according to Horwitz and colleagues, “learning to be a better listener is something that can help us all build better relationships with our pets” [(62) p. 3]. New pet adopters are also advised to learn to detect the non-verbal communication of their dogs and cats so as to adapt their actions toward them (64).

### Social identity theory

Social identity theory, the second theory of intergroup relations covered herein, is one of the most influential theories of group processes and intergroup relations (65, 66). Historically, within social psychology, social identity theory emerged as a counterpoint to the individualistic and reductionist tendencies of existing theories of intergroup relations (66). This theoretical framework has been applied to understand the relations that take place between a diversity of social groups (e.g., organizations, cultures, nations, genders). Recently, this framework has been applied to understand our relations with other animals in general (23, 67, 68).

Social identity theory accounts for the *societal and macroscopic factors* that shape the nature of the relations taking place between the members of different social groups (69). According to this theory, social groups can differ in terms of their social power, with the more powerful groups usually displaying more bias in favor of their own group (i.e., their ingroup) (70, 71). The fact that humans typically occupy a higher power position relative to their pets—given our control over their fate and a variety of valued resources (e.g., food, water, shelter, access to veterinary care)—implies that this power should be used responsibly and mindfully, rather than to our human advantage. Hence the utility of this theory for tackling, head-on, the broad sociocultural context in which human-animal relations take place, and the power dynamics these relationships involve (13).

Importantly, differences in social power can shift over time, offering hints into when and how power can change and be redistributed between the groups. For example, and based on conceptualizations of social power in social psychology, providing pets with access to decision-making opportunities and capacity for choice would indicate a gain in power. Concretely, this could take place as pets decide to go inside or outside the house thanks to a pet door, or as dogs make decisions about the routes taken during their walks. And while pet dogs are considered to be deeply enculturated in our human world (13), as indicated by their high readiness to overimitate humans (72) for example, we humans too can adjust our behaviors (e.g., organization of schedules, integration in social activities, adapting our ways of communicating) to take their preferences and perspectives into account, possibly to our mutual benefit. For example, in the context of dog training, using more hand signals (rather than relying mainly on voice) could be a way to facilitate inter-species (i.e., human-dog) communication, given that non-verbal behaviors that are typically easier to interpret for dogs compared to human voice [see McGreevy et al. (73)]. At a more structural and macroscopic level, cities can directly integrate and account for the needs of pets into their infrastructures and create public spaces dedicated to pets (i.e., dog parks, water fountains for pets).

This increase in the social power held by pets in our human societies could also be fueled by broader changes in *social norms*, another important concept in social psychology. Social norms are defined as rules and principles that are shared by group members, and that guide their behavior and determine which behaviors are acceptable or not (74). According to social identity theory, social norms are relative and context dependent (75); they can hence change depending on the social situation, and what is valued in this context and by the members of a social group. Emerging research is uncovering how social norms that are currently in flux can motivate people to change their own individual behaviors. Such *dynamic norms* are specifically defined as social norms about how other people's behavior and attitudes are changing over time (76–79). Dynamic norms have concrete consequences: For example, learning that an increasing proportion of people within one's social group (e.g., within one's country) are adopting certain behaviors (e.g., reducing their consumption of meat) motivates individual group members to adopt this behavior themselves (76).

In the realm of human-pet relations and social power *per se*, we can see how some norms are changing at the moment, in favor of pets. For example, some training methods that focus on the use of positive reinforcements have gained in popularity over the past decades (80). New legislations passed in different countries and regions also now more directly recognize pets' needs and sentience (81, 82). In the U.S., the euthanasia rates of pets have decreased over the past decades (83). More broadly, growing percentages of people are in favor of increasing our considerations toward other animals [i.e., by attributing more rights to non-human animals (84); by intending to reduce meat consumption (85)]. This general societal backdrop could also contribute to promoting more equal and mutual human-pet relations in particular. Concretely, informing people of these changes currently taking place in society, and making sure that this information is socially salient could contribute to promoting a more positive treatment of animals and pets. Pet owners could also be provided with specific facts and examples about how pet owners are increasingly providing the necessary care to their pets and have learned about their pets' needs prior to adopting them, so as to make an informed choice.

Another central concept in social identity theory pertains to the notion of *social identification*, which is defined as “that part of the individual's self-concept which derives from his or her knowledge of membership to a social group (or groups), together with the value and the emotional significance attached to it” [(86) p. 255]. This notion also provides insights into the possible solutions for achieving more mutually beneficial human-pet relations. Indeed, recent research has found that we can identify with all animals (both human and non-human animals) as a particularly broad and encompassing social group (87, 88). This work has shown that solidarity with animals—i.e., a particular dimension of identification with animals that implies feeling a strong psychological bond with, and commitment toward other animals–, predicts the tendency to have more positive and frequent contact with pets. These findings suggest that developing such a committed sense of connection to, and identification with, all animals could have beneficial repercussions for our relationships with pets in particular.

At a more concrete level, yet also building on the notion of social identification, perceiving that pets are part of the same family ingroup and that they share this social identity with us humans, could also yield more mutually beneficial human-pet relations (89). Indeed, when we share (at least) one social identity with another individual (e.g., being part of the same family), recognizing and putting forward this intersecting, ‘cross-cutting’ social identity can promote harmonious intergroup relations (90); in this case, between the members of different species. Similarly, realizing that pets are part of our communities (i.e., our neighborhood, city), and that they also share this group membership with us humans, could contribute to building more equal relations between humans and pets [for a discussion of how pets and humans form “mixed” communities (25)].

### A power reversal?

On the basis of these intergroup theories, a word of caution is in order. In the attempt to use our human power relative to pets responsibly, and to restructure human-pet relations so that they are more equal and mutual, a possible caveat should be noted, namely: the need not to create shifts in social power that would involve reallocating a disproportionate amount of valued resources to pets, to the detriment of humans (e.g., allocation of money and time). A drastic reversal in social power, whereby pets would be attributed more of our resources compared to humans, although likely to be rare, would be akin to the phenomenon of outgroup bias, which involves favoring an outgroup to the detriment of our ingroup in the way resources are distributed. While the reassignment of valued resources between social groups to favor a historically disadvantaged group has the potential to redress previous power imbalances over time (91), going too far in this vein could be detrimental to our own human agency and health, and possibly impede our capacity to care for other species (92). This would also result in yet another detrimental power imbalance, one that would favor pets over humans [e.g., a parasitic view of human-pet relations (93)].

## A self-determination theory of (human) motivation: Giving pets autonomy, opportunities for developing their competencies, as well as affection and respect

Another social psychological theory which provides insights into how we can build more harmonious and mutual human-pet relations is self-determination theory (SDT). Given its humanistic focus on individual growth and optimal development (94, 95), SDT provides a highly complementary perspective relative to the theories of intergroup relations, which focus on macroscopic social factors and how conflicts between social groups can be attenuated (88, 96). Yet when brought together, these different social psychological theories allow to cover a broad range of possible solutions for achieving more mutual and equal human-pet relations. And while SDT principles have been applied to different intergroup contexts (97, 98), they also appear particularly useful to understanding human-pet relations, as a specific type of intergroup relation.

Theoretically, SDT focuses on the dialectical relationship that operates between the individual and the social environment to understand motivation and growth. Specifically, SDT proposes that humans are geared toward becoming more self-determined and autonomous, and that provided the right social context, they can grow as individuals, master the challenges they are confronted with, and integrate new experiences so as to develop a more coherent and integrated (vs. conflicted or fragmented) sense of self (94). Importantly, these inherent tendencies will develop only to the extent that the social context (e.g., our workplace, family setting, leaders/supervisors) provides the necessary ‘nutriments’, namely by supporting the basic psychological needs for autonomy, competence, and relatedness. According to SDT, to the extent that our social contexts support these three needs, the higher our psychological wellbeing and optimal functioning should be. We argue herein that these three basic psychological needs could also be fostered and nurtured among pets specifically, to build more mutual human-pet relations. Table 1 also presents the main concepts that compose this theory.

### Promoting our pets' needs for autonomy, competence, and relatedness

Whereas SDT is a theory of human motivation, it directly relied on studies conducted with nonhuman animals to demonstrate how fundamental these needs and motivational processes are for humans *per se* (99–101). Only a few studies up to now have applied the SDT perspective to human-pet relations; these studies have found that the more pet owners report that their animal satisfies their basic psychological needs for autonomy, competence, and relatedness, the higher their psychological wellbeing (102, 103). Yet, how we, humans, can satisfy our pets' needs also represents a relevant application of SDT to human-pet relations; doing so also offer glimpses into how, specifically, mutuality can be promoted between people and their pets.

#### Autonomy

One solution could involve promoting our pets' autonomy. In SDT, the need for autonomy refers to the desire to be self-initiating in the regulation of one's actions and to be the origin of one's behaviors (104, 105). Different commentators have argued that pets have a need for autonomy and self-determination. Indeed, pets and other animals have the need and desire to exercise control in their lives and to have agency, including in decisions that have direct implications for them, such as where and how they live, as well as who they live with, and associate with (106, 107). From an animal ethics perspective, and tying back to the intergroup relations principles covered above, respect for animal agency and self-determination is “the first principle of interspecies justice” [(106) p. 231]. Such a feeling of control should also fuel higher wellbeing among animals (14). The question is: how can we interact with our pets in ways that support this autonomy?

This fundamental need could be satisfied by “empowering” our pets and providing them with opportunities to make their own decisions. Clicker training, a positive reinforcement training technique, is one concrete example to achieve this. Specifically, the “click” noise produced by the clicker is a precise way to communicate to the animal that the behavior he or she has just offered will be reinforced. The animal is a willing participant in this bonding activity and is not coerced to obey, nor does he or she fear the consequences of “being wrong.” This technique can hence be used to encourage exploration, spontaneity, and decision-making among pets. Finding ways to gather our pets' consent prior to petting them or involving them in specific activities could also directly support their need for autonomy [(107, 108), see also Ashall et al. (109) for a discussion of the notion of consent in the veterinary context]. This could be achieved by letting our pets come to us first to see if they want to be petted (or not), and by looking out for cues as to whether they want an interaction or activity to continue (or not).

As well, identifying our animal's preferences for certain activities over others, and paying attention to signs of boredom, fatigue, and over-stimulation when engaging in these activities, will directly inform us of their own preferences and tastes, as well as their strengths and limits. Doing so not only requires adequate knowledge of our pets' species- and individual-level preferences, but also necessitates, on our human end, the development of certain observational skills (110). Yet, developing such skills is likely to allow us to better identify our pets' own preferences and strengths. Importantly, such observational skills have the potential to prevent us from “projecting” our own human needs, preferences, intentions, and wishes unto our pets [i.e., anthropomorphism (111, 112)] and to better care for them.

Pets can also make choices by indicating their preferences for certain foods over others (within reason), and for the relationships they seek to maintain and develop (i.e., with specific humans and animals). They can also be trained to use tools to help us understand what they need or want (e.g., pet doorbells, talking buttons). Dogs can be given opportunities to smell during their walks and to use this highly developed sense to a greater extent [rather than having to heal continuously (113)]; they can also be given opportunities to choose the length and the pace of the walk. Again, observing our pets' responses in these situations and activities will directly inform how their need for autonomy can be supported. Of course, and as for humans, there are inherent limits to the opportunities and choices we can offer our pets, also to their own (longer-term) benefit (e.g., the types and quantity of food offered to them). We contend here that through accessible and concrete everyday actions, we can support our pets' autonomy and help them shape their own lives.

#### Competence

In SDT, the need for competence implies that individuals want to feel effective in their ongoing interactions with the social environment and experience opportunities to exercise and express their capacities (94, 101). Pets and other animals also have a need for competence. Franks and Higgins (114) have argued that both people and other animals want to be successful in attaining desired results (value effectiveness), establish what is real (truth effectiveness), and manage what happens (control effectiveness), and that wellbeing and welfare results when these three principles are simultaneously satisfied (115, 116). According to Donaldson and Kymlicka, pets want to decide what sorts of activities they learn about, engage in, and pursue mastery of Donaldson and Kymlicka (106).

Although humans have a good idea of the species-specific abilities of dogs (e.g., highly developed sense of smell, acute vision for movements), cats (e.g., great agility, night vision), and birds (e.g., superior color vision, ability to fly and to speak), these particular skills are not always developed to their full potential. Organizing activities where pets can use their specific skills would be a concrete way to fulfill their need for competence. A rather simple way to take advantage of the natural skills of pets is by setting up foraging activities instead of giving them their meal banally. The use of interactive food bools, where pets need to use their skills to access their food, also represents an accessible way to support their need for competence.

SDT research has shown that to satisfy the need for competence and to feel this sense of confidence and effectance in action, the feedback provided should be informational and positive (94). In this sense, the use of positive reinforcement techniques, such as clicker training, would also support the need for competence. Similarly, some training techniques require to breaking down a new behavior into different sequential components; doing so should also nurture our pet's competence and facilitate the attainment of goals. Identifying and setting progressive, achievable, and realistic goals for the pet, and preventing negative behaviors from occurring, also aligns with these general motivational principles. And while the expression: “setting your dog up for success” (117) synthetizes these principles and applies them specifically to dogs, many of these training principles and techniques can be used to train and enrich the daily lives of a diversity of pet species, in addition to dogs (e.g., cats, parrots, rats, hamsters, ferrets).

Another context in which the need for competence could be supported among our pets is during play, a situation often used as a learning tool, but which can be highly enjoyable both for the pet and the human (118). In play situations, pets can learn the rules about how things work and what are the boundaries, yet still have control over what will take place in these playful interactions. In this sense, enjoyable and “fair” play represents a powerful way for pets to exert and develop their sense of competence.

#### Relatedness

Finally, the need for relatedness pertains to the desire to feel connected to others, to care for and be cared for by others, and to have a sense of belongingness with other individuals and with one's community (119, 120). In interpersonal relationships, this relatedness is manifested by our involvement with another person, when showing interest in and directing energy toward this person, and by conveying that the person is significant and cared for noncontingently. Interesting, while many individuals can satisfy this need for relatedness (e.g., spouse, friends, colleagues), SDT research has typically focused on interpersonal relationships that are hierarchical and hence involve a certain imbalance in social power [e.g., teacher-student, coach-athlete, manager-worker, parent-child (121)]; yet, even in these types of relationships, relatedness can be built.

In parallel, research on human-pet relations has suggested that the relationship between companion animals (i.e., dogs) and their human caregivers bears resemblance to parent-child relationships (103, 122–124). Relatedness could be promoted in human-pet relations by being involved in the care of one's pet and by ensuring that one's pet will be cared for, regardless of constraints and external contingencies. Paying attention to one's pet, as well as demonstrating love and affection to our pet are other concrete means through which relatedness can be promoted within human-pet relations. More broadly, the need for relatedness could also be fulfilled by showing our pets respect; respect for who they are as an individual (125). Concretely, this could be achieved by ensuring that the appropriate caring behaviors, which respect the pet's needs, are integrated into one's daily routine. These caring behaviors should also be informed by the myriad of resources now available about pets and distributed *via* different sources (e.g., veterinary hospitals and organizations, SPCAs, shelters, pet products companies). Not leaving one's pet for extended periods of time is another way of caring for one's pet and providing him/her with adequate attention and presence. Again, making informed choices prior to adopting a new pet, to ensure that their needs are known and will be met continuously, despite external circumstances and contingencies, and throughout the entire life of the pet, is also crucial to maintaining relatedness.

Showing interest and paying attention to one's animal is also a challenge in a social context where productivity, achievement, and speed are highly valued, and where social media can interfere with different social activities, including walking our dogs. However, realizing how much our pets can bring a more healthy rhythm to our own lives, and facilitate our mindful attention to the present (126), could directly help us pay more attention to them, to our mutual benefit. And while the specific manners in which love and affection are displayed are likely to differ widely depending on the species of one's pet (e.g., dog, cat, rodents, fish), there are various ways to manifest affection and support to our pets (in addition to physical touch), including: the tone of voice used, our gaze, and our mere presence in the same room.

### Promoting pets' internalization of our humancentric behaviors

According to SDT and one of its sub-theories, organismic integration theory, supporting the needs for autonomy and relatedness, in particular, is critical to promote wellness and development, but supporting these needs also prompts a process called internalization. Internalization involves acquiring beliefs, attitudes, or behavioral regulations from external sources (e.g., authorities, parents, peers), and progressively transforming these external regulations into our own personal attributes, values, or regulatory styles (127). In other words, internalization involves accepting values and behaviors that were initially promoted by others by actively making them our own (128). From a motivational perspective, behaviors become internalized when they are first emitted to gain external rewards and reinforcements (i.e., as external regulations), but progressively become manifested not for the sake of attaining these external rewards, but instead because the individual perceives that the behavior allows to attain important goals (i.e., building more positive relations with others; sharing norms for acting; identified regulation). When a behavior is internalized, it becomes coherent and aligned with one's own personal values (i.e., being a good person; integrated regulation). In this sense, internalization occurs when behaviors become increasingly self-determined and emitted out of choice (129, 130).

This internalization process, we content, could also take place in human-pet relations. In this context, behaviors become internalized by pets when they are first emitted to gain external rewards and reinforcements (e.g., food reinforcers), and progressively become displayed even if these rewards are provided by humans only intermittently or replaced by secondary reinforcers (i.e., tone of voice, praise). In this sense, our human standards and norms for behaviors could, over time, become *internalized* by our pets; that is, such behaviors may become perceived as part of their life, embedded into their daily routines and habits, and also learned as ways to build a more positive relationship with their human. And let us not forget that human norms and standards for our pets' behaviors (e.g., not climbing or clawing on certain furniture, not chewing certain objects, not barking) can be seen as somewhat arbitrary from their own point of view, and may also clash with their instinctual and ‘normal’ behaviors (131). Yet, over time and given their capacity for adaptation, pets may come to learn and internalize new/alternative behaviors (e.g., climbing and clawing on the authorized climbing post; chewing on safe toys), namely those valued by us humans. In line with SDT principles, providing autonomy to, and building relatedness with, our pets could facilitate this transition toward greater self-determination and integration in daily life.

## Mutual benefits: Toward a global approach

More broadly, the current focus on building more equal and mutual relations between people and their pets aligns with a number of emerging scientific and social trends. First, the current ideas align with a general need to develop a less anthropocentric view of human-pet relations and to take full responsibility for our pets' wellbeing (13, 55), to our mutual benefit. Indeed, according to Miklosi and colleagues: “If the (human-pet) partnership is based on social competence and caring (…), then we expect (this) interaction to emerge as a result of mutual interest and on average it should have a positive effect on all partners. In other words, practice must be mutually beneficial to be considered both ethical and effective” [(20) p. 391]. Furthermore, focusing on the health and wellbeing benefits that humans derive from our relationships with pets, and assuming that these relations will automatically (and instrumentally) benefit us humans, is not warranted based on the scientific evidence (23, 57, 132). Hence the need to move beyond an instrumental view of human-pet relations, and to adopt a more interactive and integrated approach.

Second, commentators from different scientific disciplines are currently questioning the legitimacy of our beliefs in human superiority and exceptionalism (1, 110). The ideas put forward in this paper provide concrete ways for enacting an alternative vision and beliefs and building more equality, specifically in the context of human-pet relations. Recognizing that the power dynamics that operate between humans and animals are bidirectional, given that animals have also contributed to influence us humans and to shape human evolution (27, 133), may also help to deconstruct these beliefs. Third, the current ideas broadly align with the One Health approach, which explicitly recognizes the interconnections that exist between humans, animals, and nature, and how these links have direct implications for human and animal health (19). In line with this approach, the current ideas focus on the interactions and the interdependence that exist in human-pet relations specifically, and how these interactions could be structured to produce mutual benefits; in other words, how the human-pet relationship—as an interspecific interaction—can be mutualistic (134).

It should be noted that the solutions put forward herein, while theoretically-based, are by no means exhaustive. They aim to provide illustrations of what can be done concretely, and which general social psychological principles could be followed to achieve more equal and mutual human-pet relations. These solutions also seek to spark a theoretically-based reflection about the ways we can build more mutually beneficial relations between humans and pets and build bridges across scientific disciplines. We focused on one particular subgroup of animals—i.e., pets—, a subgroup with whom we have particularly close and concrete relations. While focusing on this subgroup of animals may not only be a more logistically feasible and accessible place to start to build more mutuality between humans and other animals, it could potentially serve as a springboard for questioning and changing our relationships with other types of animals (i.e., non-pet animals: wild animals, meat-animals), in line with the “pets as ambassador effect” (39, 40, 135) and the spill-over effects (secondary transfer) found in the intergroup contact literature (44, 45).

We also aimed to provide examples that apply to different species of pets. Nevertheless, the fact that many examples pertained specifically to dogs could be taken to represent a general trend in veterinary medicine and human-animal relations research, whereby dogs receive high levels of scientific attention (136). Future work should also aim to redress this imbalance, observed across pet species *per se*. Finally, while the current paper focuses on the perspective of pets, it is possible that the autonomy given to some specific species of pets (e.g., cats allowed to go outside) could impede the welfare of other animals (e.g., wild birds and small rodents). The strategies developed to provide pets with autonomy and choice should hence be mindful of such potential consequences.

## Conclusion

The current paper aimed to provide theoretically-based solutions to build more equal and mutual human-pet relations. To this aim, we first argued in favor of acknowledging the power dynamics that operate in human-pet relations and our human responsibility to care for our pets. We then applied principles from established theories in social psychology, namely theories of intergroup relations and of human motivation, to put forward possible solutions to build more mutually beneficial human-pet relations. While theories of intergroup relations allow to generate solutions at the societal level of analysis, theories of human motivation point to solutions at a more relational level. More broadly, this paper contributes to a growing recognition of the interconnections that exist between humans and other animals and the need to integrate these diverse perspectives and interests, to our mutual benefit.

## Author contributions

Both authors listed have made a substantial, direct, and intellectual contribution to the work and approved it for publication.

## Funding

This research was facilitated thank to grants from the Social Sciences and Humanities Research Council of Canada (SSHRC: 430-2018-00961) and from the Fund for Research on Health—Québec (FRSQ: 268393).

## Conflict of interest

The authors declare that the research was conducted in the absence of any commercial or financial relationships that could be construed as a potential conflict of interest.

## Publisher's note

All claims expressed in this article are solely those of the authors and do not necessarily represent those of their affiliated organizations, or those of the publisher, the editors and the reviewers. Any product that may be evaluated in this article, or claim that may be made by its manufacturer, is not guaranteed or endorsed by the publisher.
